# Expression of *Trichoderma reesei* β-Mannanase in Tobacco Chloroplasts and Its Utilization in Lignocellulosic Woody Biomass Hydrolysis

**DOI:** 10.1371/journal.pone.0029302

**Published:** 2011-12-28

**Authors:** Pankaj Agrawal, Dheeraj Verma, Henry Daniell

**Affiliations:** Department of Molecular Biology and Microbiology, Burnett School of Biomedical Sciences, College of Medicine, University of Central Florida, Orlando, Florida, United States of America; Cinvestav, Mexico

## Abstract

Lignocellulosic ethanol offers a promising alternative to conventional fossil fuels. One among the major limitations in the lignocellulosic biomass hydrolysis is unavailability of efficient and environmentally biomass degrading technologies. Plant-based production of these enzymes on large scale offers a cost-effective solution. Cellulases, hemicellulases including mannanases and other accessory enzymes are required for conversion of lignocellulosic biomass into fermentable sugars. β-mannanase catalyzes endo-hydrolysis of the mannan backbone, a major constituent of woody biomass. In this study, the *man*1 gene encoding β-mannanase was isolated from *Trichoderma reesei* and expressed via the chloroplast genome. PCR and Southern hybridization analysis confirmed site-specific transgene integration into the tobacco chloroplast genomes and homoplasmy. Transplastomic plants were fertile and set viable seeds. Germination of seeds in the selection medium showed inheritance of transgenes into the progeny without any Mendelian segregation. Expression of endo-β-mannanase for the first time in plants facilitated its characterization for use in enhanced lignocellulosic biomass hydrolysis. Gel diffusion assay for endo-β-mannanase showed the zone of clearance confirming functionality of chloroplast-derived mannanase. Endo-β-mannanase expression levels reached up to 25 units per gram of leaf (fresh weight). Chloroplast-derived mannanase had higher temperature stability (40°C to 70°C) and wider pH optima (pH 3.0 to 7.0) than *E.coli* enzyme extracts. Plant crude extracts showed 6–7 fold higher enzyme activity than *E.coli* extracts due to the formation of disulfide bonds in chloroplasts, thereby facilitating their direct utilization in enzyme cocktails without any purification. Chloroplast-derived mannanase when added to the enzyme cocktail containing a combination of different plant-derived enzymes yielded 20% more glucose equivalents from pinewood than the cocktail without mannanase. Our results demonstrate that chloroplast-derived mannanase is an important component of enzymatic cocktail for woody biomass hydrolysis and should provide a cost-effective solution for its diverse applications in the biofuel, paper, oil, pharmaceutical, coffee and detergent industries.

## Introduction

The world's energy demands are ever increasing and cannot be sustained by conventional fuel sources alone. Therefore, biofuels are needed as an alternative source of energy. The current production of fuel grade ethanol utilizes food crops such as corn grain, which consumes about 25% of U.S corn production and therefore competes with food source ([1], [2] http://www1.eere.energy.gov/biomass/pdfs/us_biofuels_industry_report.pdf). Lignocellulosic biomass is a renewable alternative source for bioethanol production, which includes agricultural wastes such as pinewood, citrus peel, corn stover, poplar waste, bagasse and rice straw. Currently, large amount of these biomass feed stocks are available for their conversion to fermentable sugars for bioethanol production (United States Department of Energy, http://www1.eere.energy.gov/biomass/feedstock_databases.html). Lignocellulosic biomass is rich in cellulose and hemicellulose which are difficult to breakdown into fermentable sugars due to the complex structure of the cell wall. For breakdown of complex biomass, chemical and physical pretreatments of these materials are necessary. These treatments are expensive, have serious environmental consequences and decrease enzymatic hydrolysis [3]. To reduce such environmental effects of pretreatments, a cost effective and environmentally friendly solution should be considered. As the cellulosic biomass is composed of complex cellulose, hemicellulose and various entangled fibers, concurrent action of different enzyme classes such as cellulases, glucosidases, hemicellulases and accessory enzymes including esterases, lipases, pectate lyases are required, in large quantities [3]. Simultaneous action of these enzymes can increase the access of each enzyme to the cellulosic biomass.

Hemicelluloses are complex polysaccharides present in plant cell wall and mannans are important constituents of hemicellulosic fraction, which are abundantly present as glucomannan or galactoglucomannan in the wood of gymnosperm plants [4]. Wood dry mass contains 20–25% of galactoglucomannan and is the main component of softwood hemicellulose. It is composed of a linear chain of D-mannopyranose and D- glucopyranose units linked by β-(1, 4) glycosidic bonds. The glucose and mannose in the linear chain are partially substituted by α-D-galactopyranosyl units via α-(1, 6) bonds. On the other hand glucomannans constitutes approximately 5% of the secondary cell wall of hardwood [5], [6]. Lignocellulosic biomass rich in mannans include softwood from gymnosperms such as pinewood (10%), poplar wood (4%), and cellulose sludge (4%) (United States Department of Energy, (http://www1.eere.energy.gov/biomass/feedstock_databases.html). Algae including *Acetabularia* and *Porphyra* contain up to 20% mannans in their cell wall [7], [8], which can be utilized for ethanol production [9]. Algae are also important producers of biodiesel, after the lipids are extracted for biodiesel production; the remaining waste is rich in carbohydrates and can be used as a substrate for bioethanol production [9].

Endo β mannanase (3.2.1.78) belongs to glycoside hydrolase enzyme family 5, which randomly cleaves β-D-1, 4-mannopyranosyl linkage in the main chain of mannans and heteromannans including galactomannans, glucomannans and galactoglucomannans. The main hydrolysis products obtained by the action of endo β mannanase are mannobiose and mannotriose [10]–[12]. Mannanases have diverse industrial applications including bleaching of the softwood pulp in paper industry, reducing the viscosity of coffee extracts rich in mannans, oil extraction of coconut meat, oil and gas well stimulation, as a stain removal agent in detergents, neutraceutical and excipient production in the pharmaceutical industry [10], [12]. Recently, mannanases have gained importance for their role in hydrolysis of the hemicellulose fraction in the lignocellulosic biomass for efficient breakdown of the complex polysaccharides into simple sugars for bioethanol production [13]. Endo β mannanase is one among the most important hemicellulases for hydrolysis of lignocellulosic biomass. Analysis of a range of enzyme combinations on palm kernel press cake (PKC) showed that including cellulases in combination with mannanase significantly improved ethanol yields up to 70 g/kg of PKC [13]. An optimal enzyme cocktail for the hydrolysis of ammonia fiber expansion (AFEX) treated dried distillers grains with solubles (DDGS) has been reported to contain high amount of mannanase. As DDGS consists of 2.5% mannans, including excess of mannanase resulted in the efficient hydrolysis of DDGS and thereby increased glucose yields [14]. Another study demonstrated that adding chimeric *Aspergillus niger* mannanase to the enzyme cocktail of *Trichoderma reesei* improved hydrolysis of lignocellulosic substrate softwood [6].

Several organisms including bacteria, actinomycetes, yeast and fungi have been reported to hydrolyze mannans. Among bacteria, Bacillus is the most established mannanase producing group and has been extensively studied [10], [15]. The most utilized fungus in the industrial production of mannanase, with immense capability to act on a variety of mannan substrates, belong to genera *Trichoderma* and *Aspergillus* [10], [16], [17]. *Trichoderma reesei* is one of the most comprehensively studied filamentous fungus which produces industrially important cellulases and hemicellulases. Endo β mannanase from *Trichoderma reesei* has been isolated, purified and characterized [16], [18], [19]. The three dimensional structure of *Trichoderma reesei* mannanase has been elucidated and reveals the presence of four disulfide bonds. Further, additional substrate binding subsites were discovered which are absent in the bacterial enzyme [20]. In another study, *Trichoderma reesei* mannanase successfully hydrolyzed galactomannan in pine kraft pulp, whereas mannanase from *Bacillus subtilis* was not able to do so [21]–[23]. Sequence alignment and hydrophobic cluster analysis have shown that mannanase from *T. reesei* consists of two modules. One is the N-terminal catalytic module and another is a C-terminal carbohydrate binding module (CBM) [19], [24]. CBM brings the enzyme in close vicinity of the polysaccharide substrate and hence increases the concentration of enzyme at the substrate [25]. A *Trichoderma reesei* mannanase mutant lacking the CBM showed five-fold less hydrolysis of ivory nut mannan when compared to mannanase with CBM [24].

Heterologous expression of both fungal and bacterial mannanase has been used for the production of enzyme via submerged fermentation. Because of the need for prohibitively expensive infrastructure for prevention of contamination by other microbes, high production cost and limited capability of fermentation facilities for producing various biomass hydrolyzing enzymes, in planta expression of these enzymes should be preferred. In planta expression of cell wall degrading enzymes have many advantages over other heterologous production systems including remarkable ability for scale up, well established large scale production and harvesting methods, increased enzyme yield/stability and various storage alternatives [26], [27]. Tobacco is a suitable host for in planta production of cell wall degrading enzymes because it produces large amount of biomass. The commercial tobacco cultivars yield up to 40 metric tons of biomass per year in three harvests [28]. Advantages of expressing biomass hydrolyzing enzymes via the chloroplast genome include high levels of expression due to thousands of transgene copies in each cell, containment of transgenes via maternal inheritance and minimal pleiotropic effects due to compartmentalization of enzymes within chloroplasts, away from the cell wall.

Several reports have investigated the heterologous production of biomass degrading enzymes in plants via nuclear transformation [29], [30]. Production of enzymes through nuclear transformation has several limitations including low expression levels, gene silencing and position effect [31]. On the contrary, plastid transformation has the ability to accumulate large amount of foreign proteins (up to 72% levels of total leaf protein) [32], [33]. Engineering foreign genes in the chloroplast genome may provide containment from pollen transmission as organelle genes are maternally inherited in most crops [34]. In addition, harvesting leaves before flowering provides nearly complete transgene containment [31], [34]. Transgene integration into the chloroplast genome occurs by site specific homologous recombination; therefore, there is no gene silencing or position effect [31]. Proper protein folding and disulfide bond formation occurs in chloroplast; hence the expressed protein is properly folded and fully functional [35]–[38]. Also, compartmentalization within chloroplasts minimizes negative pleiotropic effects of cell wall hydrolyzing enzymes [39] or even increases biomass [40] except in one report where expression of biomass degrading enzyme had drastic phenotypic effect on the transplastomic plants [41]. Moreover, cost of production of enzymes in tobacco chloroplasts is significantly reduced. A recent study reported that about 64 million units of pectate lyase and 10,751 million units of endoglucanase can be produced per year per acre of tobacco; therefore, enzyme production cost was 3100 and 1480 fold less when compared to their commercial counterparts produced via fermentation [42]. Recent NC State University Tobacco Guide 2011 estimates the cost of production of tobacco as $3169/acre.

A chloroplast-derived enzyme cocktail has been formulated for the hydrolysis of lignocellulosic biomass based on its composition [42] but did not contain mannanase which is an integral part of an enzyme cocktail for biomass hydrolysis [14]. Woody biomass including pinewood and algal biomass consists of significant amount of mannans. Therefore, in this study the *man*1 gene from *Trichoderma reesei* was expressed into tobacco chloroplasts. The chloroplast-derived mannanase was characterized and used to formulate an enzyme cocktail for pinewood hydrolysis. Use of mannanase in enzyme cocktail released 20% more fermentable sugars from pinewood than using cocktail without mannanase. To our knowledge, this is the first report of expression of fungal mannanase in plants and their direct utilization in enzyme cocktails, for lignocellulosic biomass hydrolysis without any need of purification.

## Results

### Construction of chloroplast transformation vector harboring *man*1 gene

**Figure 1:**
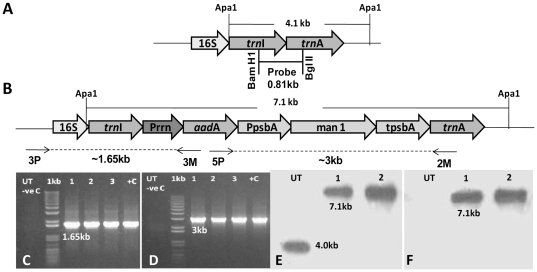
Characterization of transplastomic plants. A & B, Schematic representation of chloroplast flanking sequences used for homologous recombination, probe DNA sequence (0.81 kb), primer annealing sites (3P/3M and 5P/2M) and expected products of untransformed and transgenic lines when digested with *Apa*I. Prrn, rRNA operon promoter; *aad*A, aminoglycoside 3′ adenylyltransferase gene; PpsbA, promoter and 5′ untranslated region of the *psb*A gene; tpsbA, 3′ untranslated region of the *psb*A gene. C, PCR analysis using primer pairs 3P/3M and D, 5P/2M. Lanes 1–3, transplastomic lines; UT, Untransformed (−ve C); +C, positive control for 3P/3M confirmed established transplastomic line, for 5P/2M pLD man1; 1 kb, 1 kb plus DNA ladder. E, Southern blot hybridized with the flanking sequence probe. Lanes 1–2, transplastomic lines; UT, Untransformed. F, Southern blot hybridized with *man*1 probe. Lanes 1–2, transplastomic lines; UT, Untransformed.

Coding sequence of *man*1 gene (three exons) was amplified by PCR [43] from *Trichoderma reesei* genomic DNA. Agarose gel analysis of the final PCR product showed a product of ∼1338 bp, which was cloned in pCR Blunt II Topo vector (Invitrogen) and sequence was verified. Tobacco chloroplast transformation vector pLD-*man*1 (Figure 1B) was constructed with *man*1 coding sequence based on the universal chloroplast vector that targets the transgene expression cassette into the transcriptionally active spacer region between the *trn*I and *trn*A genes (Figure 1A) of the chloroplast genome for integration via homologous recombination [44]. The *man*1 gene was driven by light and developmentally regulated *psb*A promoter and 5′ UTR, which contains several ribosome binding sites to enhance transgene expression levels [32]. The 3′ UTR located at the 3′ end of *man*1 coding sequence stabilized the transcript. The *aad*A gene conferring spectinomycin resistance for selection of transformants was driven by the constitutive tobacco plastid ribosomal operon promoter (*Prrn*).

### Evaluation of site-specific integration and homoplasmy of transplastomic plants

Transplastomic mannanase plants were regenerated as described previously [45]. Six independent shoots (per 10 bombardments) appeared from the leaves placed on the regeneration medium containing spectinomycin within 3–6 weeks after bombardments with pLD-*man*1 plasmid DNA coated on gold particles. PCR analysis using 3P/3M validated the site-specific integration of the transgenes into the tobacco chloroplast genome. The 3P primer lands on the native chloroplast genome within the 16S *rRNA* gene upstream of the gene cassette and 3M primer lands on the *aad*A gene which is located within the gene cassette (Figure 1B). PCR reaction with 3P/3M primers generated a 1.65 kb PCR product in transplastomic lines (Figure 1C, Lanes: 1–3), which should be obtained only if site-specific integration had occurred. Nuclear transformants, mutants or untransformed plants did not show any PCR product as 3P or 3M primer will not anneal (Figure 1C, Lane: UT). The integration of *aad*A and *man*1 genes was verified by using 5P and 2M primer pair for PCR analysis. These primers anneal at different locations within the transgene cassette. The 5P primer anneals to the *aad*A gene whereas 2M primer anneals to the *trn*A coding sequence (Figure 1B). The use of 5P/2M primer pair produced a PCR product of ∼3 kb in the transplastomic lines and positive control (pLD-*man*1) whereas untransformed plant did not show any product (Figure 1D). After PCR analysis, transplastomic plants were moved to additional two rounds of selection (second and third) to achieve homoplasmy.

Southern blot analysis was performed to determine homoplasmy and to further confirm the site specific integration. The flanking sequence probe (0.81 kb, Figure 1A), which hybridizes with the *trn*I and *trn*A genes allowed determination of homoplasmy or heteroplasmy and site-specific integration of the transgene cassette into the chloroplast genome. Hybridization of nylon membrane with flanking sequence probe produced fragments of 7.1 kb in transplastomic lines (Figure 1E, Lanes 1 & 2) and 4.0 kb (Figure 1E, Lane UT) in untransformed plant. Absence of 4 kb fragment in transplastomic lines confirmed homoplasmy (within the detection limits of Southern blot) and stable integration of foreign genes into the chloroplast genome, whereas the detection of 4 kb fragment in untransformed plants confirmed that these plants lacked foreign genes. In addition, the *man*1 probe was utilized to verify the presence of *man*1 gene, which produced a 7.1 kb fragment in transplastomic lines (Figure 1F, Lanes 1 & 2). No hybridizing fragment was observed in the untransformed line confirming the absence of the *man*1 gene (Figure 1F, Lane UT).

### Phenotypic evaluation of mannanase plants

**Figure 2:**
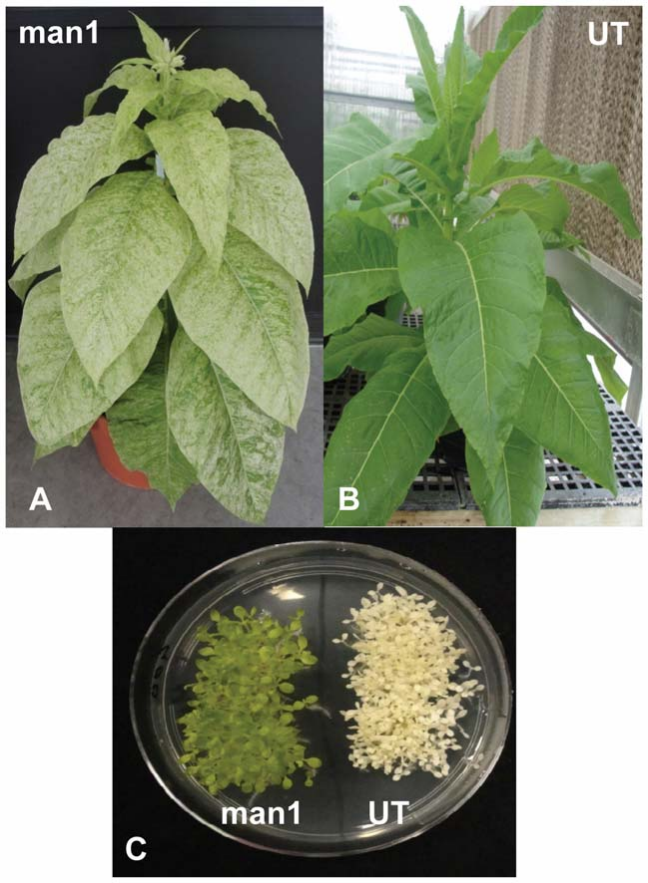
Phenotype of transplastomic mannanase plants. A, Mannanase transplastomic plant growing autotrophically in the green house. Mannanase plants were fertile and set seeds. B, Untransformed (UT) plant C, Transplastomic (man1) seeds and Untransformed (UT) seeds germinated on MSO medium containing spectinomycin (500 mg/l) showing lack of Mendelian segregation.

Homoplasmic lines were transferred to Jiffy pellets and were kept in high humidity conditions for 2 weeks before being transferred to the green house to grow under autotrophic conditions. Mannanase transplastomic plants showed mild phenotypic effects in green house with some leaves turning pale as they matured. In spite of this, transplastomic plants grew normally, flowered, set seeds and produced biomass similar to untransformed plants (Figure 2A and 2B). Mannanase T1 seeds were germinated along with untransformed seeds on spectinomycin (500 mg/l) selection medium. Mannanase seedlings remained green whereas untransformed seeds turned white (Figure 2C). These results, observed among several hundred seedlings (only one representative plate shown in Figure 2C), indicate that the transgenes were inherited to the progeny without Mendelian segregation.

### Evaluation of chloroplast-derived mannanase enzyme activity

**Figure 3:**
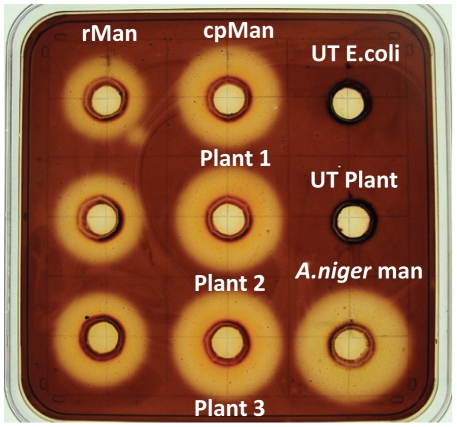
Gel diffusion assay for mannanase activity. Agar plate with 0.1% locust bean gum substrate stained with Congo red dye to evaluate mannanase activity. rMan, 100 µg of *E.coli*-derived mannanase crude extract; cpMan 100 µg of leaf extract from different transplastomic plant lines (Plant 1, 2 & 3); UT *E.coli*, Untransformed *E.coli* extract; UT plant, Untransformed plant extract; *A.niger* man, purified *Aspergillus niger* mannanase (Megazyme).

Qualitative gel diffusion assay using Congo red dye was performed in order to assess the enzyme activity of chloroplast-derived mannanase (cpMan) and *E.coli*-derived mannanase (rMan). The mannanase enzyme breaks down the polymeric galactomannan substrate, reducing the binding of Congo red dye and consequently generates a clearing zone. Both cpMan and rMan showed visible zone of clearance around the wells indicating gel areas hydrolyzed by endo-β-mannanase activity (Figure 3). Mannanase enzyme activity was directly proportional to the diameter of the zone of clearance. Moreover, the diameter of the clearing zone in cpMan was more than rMan when equal amount of protein crude extract (100 µg) was loaded in these wells indicating that cpMan was more active than rMan. No clearing zone was observed in untransformed plant extract and *E.coli* harboring pLD vector (without *man*1 gene, Figure 3). Circular area hydrolyzed by commercial purified mannanase (endo-β-mannanase from *Aspergillus niger*, Megazyme) was also clearly visible surrounding the well. Furthermore, in blank wells without the substrate, none of the extracts showed any clearing zone or non specific activity.

### Characterization of chloroplast-derived and *E.coli*-derived mannanase

**Figure 4:**
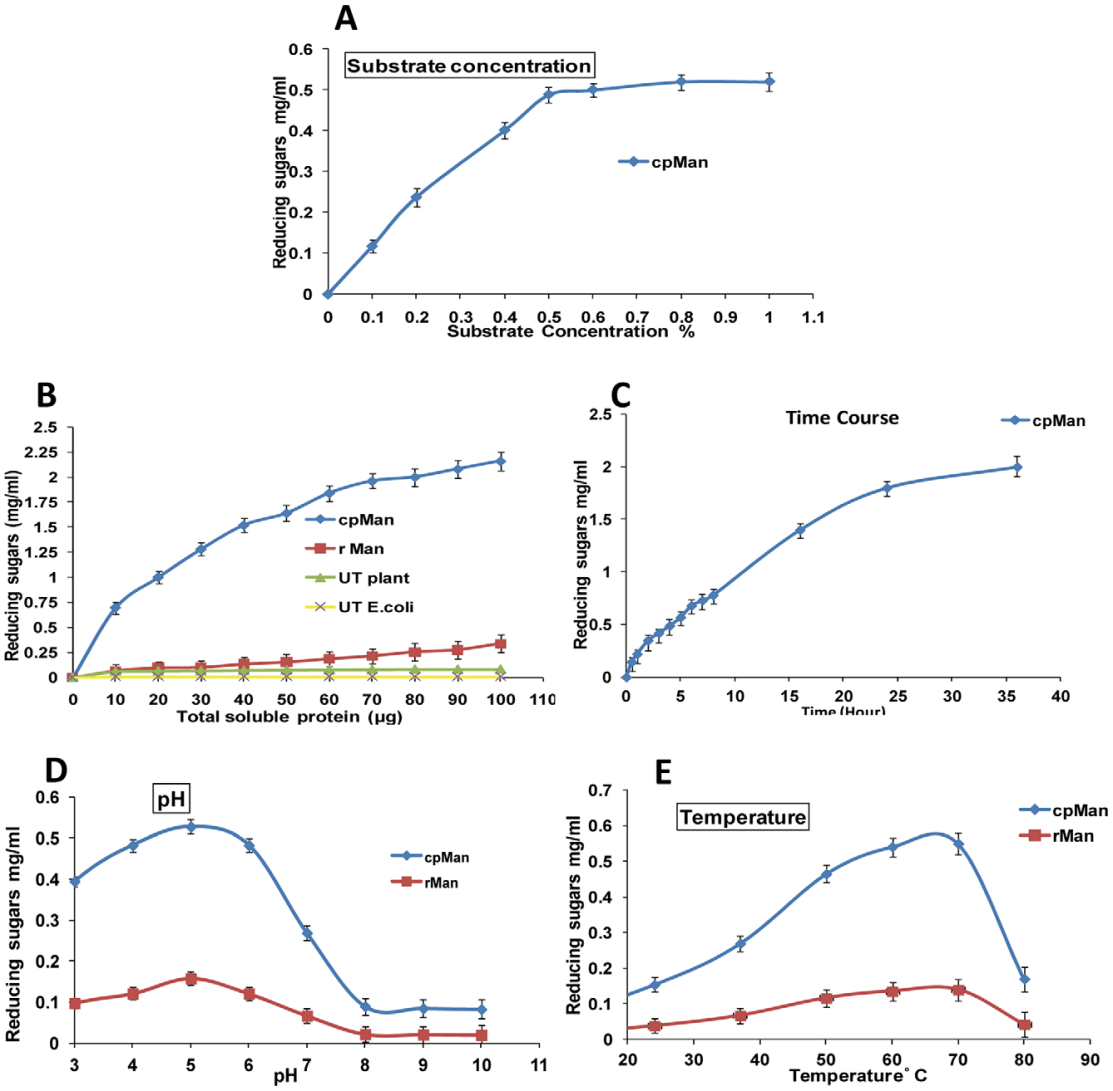
Characterization of chloroplast-derived mannanase. A, Effect of increasing locust bean gum concentration on cpMan activity. B, Substrate locust bean gum (0.5%) incubated with crude enzyme extracts in increasing concentrations of total soluble protein at 70°C, pH 5.0 in a reaction for 16 hours. C, Effect of incubation time on cpMan activity. D, Effect of pH on cpMan and rMan activity. E, Effect of temperature on cpMan and rMan activity. 30 µg of total soluble protein was incubated with 0.5% of locust bean gum at indicated reaction parameters for 2 hrs. rMan, *E.coli*-derived mannanase crude extract; cpMan, leaf extract from transplastomic plants; UT *E.coli*, Untransformed *E.coli* extract; UT plant, untransformed plant extract. (Error bars indicates the standard deviation; n = 3).

Since chloroplast promoters function efficiently in *E.coli*, crude extract from *E.coli* harboring pLD-man1 was used for quantitative comparison of enzyme activity with chloroplast-derived enzyme. Enzyme assays were performed using locust bean gum (galactomannan) as the substrate. Both plant and *E.coli* extract showed optimal activity at 0.5% locust bean gum and reducing sugars increased at a directly proportional rate until this concentration was reached (Figure 4A). Hence, all subsequent enzyme characterization studies were carried out at this substrate concentration. Both cpMan and rMan released more reducing sugars with increasing protein concentration. However, chloroplast-derived mannanase released more reducing sugars at all of the tested total soluble protein (TSP) concentrations when compared to the *E.coli*-derived mannanase (Figure 4B). This data shows that the chloroplast expression system is more efficient and showed up to 6–7 folds higher end point reducing sugar at 100 µg TSP than the bacterial system. Untransformed plant extract and *E.coli* did not yield any significant amount of reducing sugars under standard assay conditions. The primary purpose of this study was to use the plant crude extract without purification for lignocellulosic biomass hydrolysis in order to make the process cost-effective. Time dependent changes in enzyme activity of cpMan showed a linear increase in release of reducing sugars with increasing time. The cpMan continued to increase enzyme activity even up to 36 hours of incubation indicating stability of this enzyme for long durations at 70°C (Figure 4C).

The primary purpose of this study was to use crude extracts of chloroplast-derived mannanase along with the other chloroplast-derived enzymes in a cocktail for hydrolysis of lignocellulosic biomass. Temperature and pH are important characteristics for efficient use of crude enzyme extracts in cocktails. Crude enzyme extract (30 µg TSP) from plant and *E.coli* harboring mannanase expression cassette was used to study the effect of pH and temperature on mannanase activity using the locust bean gum (0.5%) as substrate. The optimal pH for cpMan and rMan under the standard assay conditions was 5.0. The pH stability curve showed that cpMan retained >50% of its maximal activity within a broad pH range from pH 3 to pH 7, whereas rMan retained only 42% within this range. However, at pH≥8, both cpMan and rMan lost more than 80% of its activity (Figure 4D). These data suggest that mannanase enzyme was more active in the acidic pH range. The optimal temperature for cpMan and rMan was 70°C under the standard assay conditions. The enzyme activity increased with increase in temperature up to 70°C in both *E.coli* and chloroplast-derived mannanase as indicated by the temperature stability curve. Further rise in temperature affected enzyme activity drastically and about 70% of its activity was lost at 80°C (Figure 4E).

Young, mature and old leaves from transplastomic mannanase plants were collected and mannanase activity was measured using carob galactomannan as the substrate. One unit of mannanase activity is defined as the amount of enzyme which released one micromole of reducing-sugar equivalents per minute from low viscosity carob galactomannan (2 mg/ml) at pH 5.0 and temperature 70°C. Maximum enzyme activity was observed in mature and old leaves (25 Units/g fresh weight) of transplastomic mannanase plants, where as young leaves showed 44% less activity (11 Units/g fresh weight). No mannanase activity was detected in untransformed crude leaf extracts whereas *E.coli*-derived mannanase had 6–7 folds less activity when compared to chloroplast-derived mannanase at 100 µg TSP. Based on the observed expression levels, up to 2,366 units of mannanase can be harvested from each tobacco plant. With 8000 tobacco plants grown in one acre of land, 18 million units of mannanase can be produced per single cutting. Typically with 3 cuttings per year 56 million units of mannanase can be harvested per year. These results were obtained using an experimental Petite Havana variety of tobacco which gives about 2.2 ton biomass of fresh leaves. The commercial cultivar produces 20 times more biomass, hence it is expected to provide 20 fold higher enzyme yield.

### Enzyme cocktail for hydrolysis of pinewood

**Figure 5:**
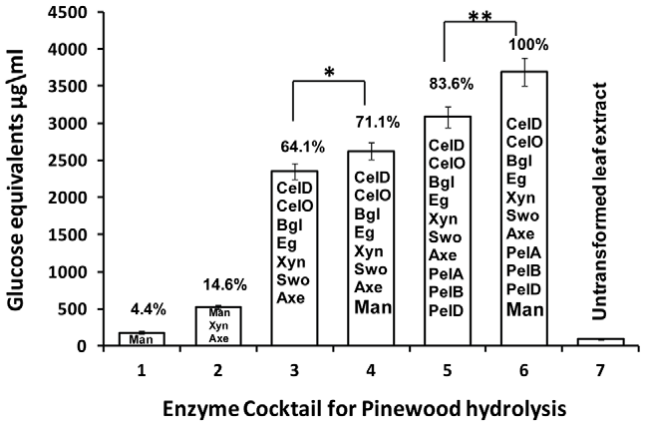
Enzyme cocktail for pinewood hydrolysis. Pinewood (200 mg/5 ml) hydrolysis using different formulations of crude enzyme cocktails. Glucose equivalents released were quantified using DNS method. 200 ug TSP of crude chloroplast derived enzyme extracts were used. Man, Mannanase; Xyn, Xylanase; Axe, Acetyl xylan esterase; CelD, Endoglucanase; CelO, Exoglucanase; Bgl, β glucosidase; Eg1, Endoglucanase; Swo, Swollenin; Pel A, B, D, Pectate lyase. (Error bar indicates standard deviation among triplicates, * p value = 0.038, ** p value = 0.013, p value were calculated using t- test).

Chloroplast-derived enzymes were used in different formulations to make various cocktails for hydrolysis of pinewood. Chloroplast-derived mannanase (Man) alone showed 4.4% of the total hydrolysis of pinewood (Figure 5, bar 1). The hydrolysis was further increased up to 14.6% when mannanase was mixed with xylanase (Xyn) and acetyl xylan esterase (Axe; Figure 5, bar 2). When we used Xyn and Axe along with endoglucanases (CelD, Eg1), exoglucanase (CelO), β glucosidase and swollenin (Swo), the hydrolysis increased up to 64.1% (Figure 5, bar 3). Supplementation of Man to this cocktail enhanced the hydrolysis by 11% attaining 71.1% of the total hydrolysis (Figure 5, bar 4). Besides cellulose and hemicellulose, pectin is the core structural component of plant cell wall of woody plants which includes pine trees. Hydrolysis of pectin component should therefore increase the release of fermentable sugars by cellulases and hemicellulases. When we treated pinewood with pectate lyases (PelA, PelB, PelD) followed by supplementation with the enzyme cocktail in bar 3, the overall hydrolysis was extended up to 83.6% (Figure 5, bar 5). Addition of mannanase to this cocktail boosted the hydrolysis to maximum amount resulting in liberation of 20% more glucose equivalents (Figure 5, bar 6). Statistical analysis between cocktails with (Figure 5, bar 4 and 6) or without mannanase (Figure 5, bar 3 and 5) showed significant difference in release of fermentable sugars. Addition of leaf extract from untransformed plants to pinewood did not yield any measurable sugars (Figure 5, bar 7). These results indicate that mannanase plays a significant role in efficient hydrolysis of pinewood biomass to release fermentable sugars.

## Discussion

Lignocellulosic biomass is composed of complex, heterogeneous intertwined polymers. Therefore, concurrent presence of different classes of cell wall degrading enzymes that can disintegrate biomass and increase the access of each other to the complex structure of biomass is required for their efficient hydrolysis to release fermentable sugars. Thus, a mixture of enzymes such as cellulases, hemicellulases including mannanases, ligninases and accessory enzymes like lipases, pectate lyases, esterases may be required, depending upon the composition of the biomass [3]. In this study, fungal mannanase was expressed in tobacco chloroplasts. To our knowledge, this is the first report of expression of fungal mannanase in plants and its direct utilization in enzyme cocktails for lignocellulosic biomass hydrolysis. For production of mannanase, tobacco plants harboring the *man*1 gene from *Trichoderma reesei* were generated. Site-specific integration of mannanase gene into the chloroplast genome was achieved by using the transcriptionally active spacer region between the *trn*I *and trn*A genes for homologous recombination. This region has been consistently used for efficient transgene integration and has several unique advantages [32], [46], [47]. We used the *psb*A promoter and 5′ UTR to achieve high levels of expression. The endogenous *psb*A regulatory elements have been used for the creation of transplastomic tobacco plants with elevated expression levels for a large number of diverse proteins [32], [33], [45], [48]. The transplastomic mannanase plants exhibited maternal inheritance of transgenes. In most crop species, organelle genomes are maternally inherited thereby excluding plastid integrated transgenes from pollen transmission. During pollen development plastids are unequally distributed, localizing all plastids into vegetative cells and excluding generative cells. Hence, sperm cells which originate from generative cells lack plastids [34]. Therefore, transplastomic tobacco plants lack Mendelian inheritance. Maternal inheritance of transgenes was demonstrated in transplastomic tobacco plants where only 6 out of 2.1 million seedlings showed paternal inheritance with a frequency of 2.86×10^−6^ [49].

Several enzymes have been expressed in chloroplasts for biomass hydrolysis to release fermentable sugars. Most of these enzymes are non-toxic to plant cells because of their compartmentalization within chloroplasts [39], [40], [42], whereas in one of the report high level expression of cell wall degrading enzymes in tobacco chloroplasts had severe phenotypic effects [41]. The observed phenotype had been attributed to the presence of carbohydrate binding module (CBM) in the cellulolytic enzymes. CBM plays an important role in binding of the enzyme to the carbohydrate substrate. Celluloytic enzymes when expressed in chloroplasts might cause deleterious effect by sequestration or degradation of the intermediates of carbohydrate metabolism [41]. We also observed mild phenotypic effects in mannanase transplastomic plants, which could be due to the presence of CBM in mannanase. Mannanase from *Trichoderma reesei* has been reported to contain a carbohydrate binding module, which increases its hydrolytic activity [24]. The observed phenotypic effect could also be due to the decreased chloroplast thylakoid lipid content in transplastomic plants expressing mannanase. Our preliminary studies showed decrease in monogalactosyldiacylglycerol (MGDG) and digalactosyldiacylglycerol (DGDG) in transplastomic lines expressing mannanase (data not shown) and further investigations are in progress to understand the observed phenotype. An Arabidopsis mutant deficient in DGDG showed similar phenotype [50], [51]. In our study, we used the *psb*A promoter from tobacco for hyper- expression. Use of a heterologous *psb*A promoter [32] or gene 10 regulatory elements [31] could lower the expression levels and produce mannanase plants without such phenotypic effects.

Qualitative gel diffusion assay with locust bean gum (galactomannan) substrate using Congo red dye showed endo β mannanase activity in crude extracts of transplastomic mannanase plants and *E.coli*. Similar assay has been used for detection and quantification of endo-β-mannanase activity present in seeds, fruit, bulbs and fungi [52], [53]. Congo red shows high specificity of binding for polysaccharides containing adjacent (1, 4) β-linked D-glucopyranosyl units and galactoglucomannans [54]. Endo mannanase activity lessens the oligomeric length and hence decreases binding of Congo red dye. The assay is insensitive for exo-activity and therefore confirms specific endo-mannanase activity. The action of mannanase therefore creates a clearing zone, which is proportional to the enzyme concentration. This confirms that the crude leaf extract from transplastomic plants contains active mannanase. Since the 3D structure of mannanase consists of disulfide bonds, proper folding of the protein is important for a fully functional enzyme. Chloroplasts have the ability to perform post-translational modifications such as disulfide bond formation, assembly of multimers and lipid modifications [36], [40]–[42], [46], [55]–[57]. Chloroplast-derived mannanase folded correctly and was fully functional. Lack of disulfide bond formation in *E.coli* might be the reason for the low activity of mannanase expressed in *E.coli*, when compared to chloroplast-derived enzyme. Also in a recent study, inhibitors were identified in crude *E.coli* extracts because addition of *E.coli* crude extract to plant extracts significantly decreased the enzyme activity in the plant extracts [42].

In the current study, 25 units of mannanase were obtained per gram fresh weight of mature leaves harvested at 6PM. Chloroplast-derived mannanase had 6–7 fold higher mannanase activity than *E.coli* mannanase. Higher activity (up to 24 fold) in chloroplast-derived biomass hydrolysis enzymes (CelO, Eg1) when compared to those expressed in *E.coli* was reported previously [42]. Characterization of chloroplast expressed mannanase showed that the enzyme is biologically active as the fungal enzyme in the pH range of pH 3.0 to 7.0 with the peak activity at pH 5.0. The optimal temperature for the chloroplast-derived enzyme was 70°C. Such high temperature appears to be common with fungal β mannanase [16], [18], [58]. As chloroplast expressed mannanase was functional in crude enzyme extracts derived from mannanase expressing plants, it can be directly added to an enzyme cocktail for biomass hydrolysis without the need for any purification, thereby lowering the cost. According to NC State University Tobacco guide 2011, the cost of tobacco cultivation is $3169 per acre. Based on the observed expression levels of mannanase, about 56 million units of mannanase can be produced per acre per year of tobacco cultivation with the production cost as low as 0.005 cents per enzyme unit (as defined in the commercial source Megazyme). This cost is 6,000 fold less when compared with the commercial purified mannanase (Megazyme).

In softwood like pinewood, glucomannans are closely associated with cellulose microfibrils and are integrated into the mass of cellulose. These glucomannans are arranged in parallel to cellulose fibrils and are tightly interconnected [6], [59]. This structural arrangement could inhibit the access of cellulases to the cellulose fibrils. Further in a recent study, it was reported that mannan polysaccharides are masked by pectic homogalacturonan (HG) in the primary cell wall and the recognition of mannan epitopes was greatly increased by enzymatic removal of pectic HG by treatment with pectate lyases [60]. Such type of association (indicated by the masking of mannans) may have a structural role in maintaining primary cell wall integrity. Also, pectic HG may coat mannans and other hemicelluloses, and thus limit or control the access of enzymes to these polysaccharides [60]. Therefore, for the efficient breakdown of softwood biomass, an enzyme cocktail comprising of mannanase and other cellulolytic enzymes are required. In our study, when mannanase was added to two different cocktails (Figure 5 bar 3 and bar 5) hydrolysis was enhanced significantly (Figure 5 bar 4 and bar 6). This could be due to the hydrolysis of the mannans present in the pinewood resulting in loosening of the structural arrangement and increased access of cellulases, thereby resulting in enhanced glucose release. It is well known that carbohydrate binding module (CBM) binds to the carbohydrate, increases the enzyme concentration at the substrate surface and augments the effectiveness of enzyme [25].

Low cost production of mannanase would be highly beneficial for its diverse applications in the biofuel, paper, oil, pharmaceutical, coffee and detergent industries. Expression of mannanase in plant chloroplasts is an important addition to the list of different cellulolytic enzymes expressed in chloroplasts, which significantly enhances the release of fermentable sugars from the lignocellulosic biomass. This study reports the first successful expression of fungal mannanase in plants and its utilization in the release of fermentable sugars for bioethanol production.

## Materials and Methods

### Construction of chloroplast transformation vector harboring *man*1 gene

*Trichoderma reesei* genomic DNA was obtained from ATCC and used as template for the amplification of three exons of mannanase gene (L25310) using sequence specific primers. Full length coding sequence of mannanase was amplified from the exons by a PCR based method [43] using the forward of first exon flanked by *Nde*I restriction site and reverse of third exon flanked by *Xba*I restriction site. Full length amplified product was ligated to pCR Blunt II Topo vector (Invitrogen) and checked for any PCR errors by DNA sequencing (Genewiz). Mannanase coding sequence was excised from Topo vector by double digestion with *Nde*I and *Xba*I and inserted into the pLD vector [36], to create the tobacco chloroplast expression vector. The final clone was designated as pLD-*man*1.

### Generation of transplastomic tobacco plants

*Nicotiana tabacum* var. Petite Havana was grown aseptically on Murashige and Skoog medium containing 30 g/l sucrose. Sterile young leaves from plants at 4–6 leaf stages were bombarded using gold particles coated with plasmid DNA of chloroplast transformation vector pLD-*man*1 using Bio-Rad PDS-1000/He particle delivery system as described earlier [45], [46]. Bombarded leaves were kept in dark for 48 hours, cut into small pieces and then placed on RMOP regeneration media containing 500 mg/l spectinomycin for selection of transformants [45], [46]. Putative transplastomic shoots emerged in 4–6 weeks after selection.

### Confirmation of site-specific integration of transgenes by PCR analysis

Putative transplastomic shoots were screened by PCR for transgene integration. Total plant DNA was extracted from the putative transplastomic shoots using Qiagen DNeasy Plant mini kit following manufacturers' protocol and used as template for PCR analysis. To verify the site-specific integration of the transgene cassette into the *trn*I/*trn*A inverted repeat region of chloroplast genome, PCR was carried out using the primer sets 3P-3M (3P- 5′-AAAACCCGTCCTCAGTTCGGATTGC-3′ and 3M- 5′CCGCGTTGTTTCATCAAGCCTTACG-3′) and 5P-2M (5P- 5′CTGTAGAAGTCACCATTGTTGTGC-3′ and 2M- 5′-TGACTGCCCACCTGAGAGCGGACA-3′) as described earlier [36], [45]. Primer 3P anneals to the native chloroplast genome upstream of the site of integration and primer 3M is complimentary to the *aad*A gene. Primer 5P anneals with the *aad*A gene whereas primer 2M is complimentary to the *trn*A gene. PCR amplification was carried out using following program – Initial denaturation at 94°C for 5 minutes; 30 cycles of 94°C for 1 minute, 56°C for 1 minute, 72°C for 3 minutes; final extension at 72°C for 10 minutes [45]. Amplified products were analyzed by agarose gel electrophoresis. After the confirmation of site-specific integration of transgene cassette, leaves were cut into small pieces and placed on RMOP media containing spectinomycin 500 mg/l for second round of selection. Subsequently, the regenerated shoots were rooted on half strength MS medium containing spectinomycin 500 mg/l for third round of selection and evaluated for homoplasmy by Southern blot.

### Confirmation of homoplasmy by Southern blot analysis

Total plant genomic DNA was isolated from PCR confirmed shoots and digested completely with *Apa*I (NEB) enzyme. The digested DNA was separated on 0.8% agarose gel and placed in depurination solution (0.25 N HCl) for 15 minutes followed by two washes with double distilled water for 5 minutes each. The gel was then soaked in transfer buffer (0.4 N NaOH, 1 M NaCl) for 20 minutes and blotted onto the nylon membrane. The membrane was rinsed twice in 2× SSC (0.3 M NaCl and 0.03 M Sodium citrate) and the DNA was cross-linked to the membrane using GS Gene linker UV chamber. The 0.81 kb flanking sequence for probe was generated by double digestion of pUC-Ct vector with *Bam*HI and *Bgl*II. The mannanase exon1 DNA fragment was amplified and used for gene specific probe preparation. The DNA fragments for probe were labeled with ^32^P α [dCTP] using Ready-to-go DNA labeling beads (GE) following manufacturer's protocol. The membrane was hybridized with the labeled probe using Stratagene Quick-HYB hybridization solution following manufacturer's instructions. After hybridization, the membrane was exposed overnight to X-ray film with an intensifying screen at −80°C and then developed to visualize the autoradiographic signal to confirm homoplasmy. The homoplasmic shoots were transferred to autotrophic medium, kept in high humidity for 2 weeks and then transferred to the green house as described earlier [45]. Transplastomic seeds were surface sterilized and placed on half strength MSO medium containing spectinomycin (500 mg/l) along with untransformed seeds.

### Crude enzyme preparation from *E. coli*

*E.coli* cells (XL-10 gold) harboring pLD-*man*1 vector and pLD vector (without *man1* gene) were grown overnight at 37°C in LB media containing 50 mg/l ampicillin. The cells were collected by centrifugation at 8,000 rpm under cold conditions and washed with 50 mM sodium citrate buffer (pH 5.0). The cells were finally suspended in 3 ml of 50 mM sodium citrate buffer (pH 5.0) containing protease inhibitor cocktail (Roche), followed by 5 sonication pulses of 30 seconds each with pause time of 30 seconds between the pulses. The supernatant was collected by centrifuging the lysate at 10,000 rpm under cold conditions and used as crude *E.coli* enzyme extract in the functional assays. Protein concentration in the extract was quantified using Biorad protein assay kit (Bio-Rad) based on the method of Bradford following manufacturer's protocol.

### Enzyme preparation from transplastomic tobacco leaves

Fresh leaves were harvested from green house grown transplastomic mannanase plants along with untransformed plants and were ground in liquid nitrogen. The ground material was suspended in 50 mM sodium citrate buffer (pH 5.0) containing protease inhibitor cocktail (Roche) and vortexed for 15 minutes at 4°C. The supernatant was collected by centrifugation at 10,000 rpm under cold conditions. The extract was filtered through 0.2 µm syringe filter followed by Amicon Ultra centrifugal filter unit-4 (10,000 NMWL) to remove the sugars present in the extract. This extract was used as crude plant enzyme extract for functional assays. Protein concentration was determined (mg/ml) using Biorad protein assay kit (Bio-Rad) based on the method of Bradford following manufacturer's protocol.

### Mannanase gel diffusion assay

Gel diffusion assay was performed to evaluate the mannanase activity in chloroplast-derived (cpMan) and *E.coli*-derived (rMan) crude extract using locust bean gum (Sigma G0753). Locust bean gum is galactomannan extracted from seeds of *Ciratonia siliqua*. Locust bean gum (0.1%) was suspended in 50 mM sodium citrate buffer pH 5.0 by boiling while constantly stirring. The mixture was centrifuged at 3,000 rpm and the supernatant was collected. Phytagel (0.7% w/v) was dissolved in this mixture by heating. The contents were then poured into plates and were allowed to set. Wells were punctured into the gel plates. Crude enzyme extract cpMan and rMan (100 µg) were added into the wells along with *Aspergillus niger* mannanase (Megazyme) as positive control, whereas protein extract from untransformed plant and *E.coli* harboring pLD vector (without man1 gene) as negative controls. The plates were incubated at 37°C for 16 hours. These plates were then shaken gently for 15 minutes after adding Congo red dye (1% w/v) and washed with 1 M NaCl until the wells were transparent [52]. The zone of clearance showing mannanase activity was investigated.

### Determination of optimal substrate concentration, pH and temperature for mannanase activity

Optimal substrate concentration was determined by using different concentrations of locust bean gum substrate ranging from 0.1% to 1% in a reaction containing 30 µg TSP of cpMan. The effect of temperature on mannanase activity was investigated by incubating 30 µg TSP of cpMan and rMan with 0.5% locust bean gum (pH 5.0) at different temperatures of 24, 37, 50, 60, 70, 80°C. For evaluation of the optimal pH, a reaction with 30 µg TSP of cpMan and rMan with 0.5% of substrate was setup in sodium citrate buffer (pH 3.0, 4.0, 5.0 and 6.0), phosphate buffer (pH 7.0 and 8.0) and Tris-HCl buffer (pH 9.0 and 10.0) at 70°C for 2 hours. To determine the stability of cpMan for longer duration, reactions were set with 0.5% substrate containing 30 µg TSP of cpMan at 70°C for different time points ranging from 30 minutes to 36 hours.

### Mannanase enzyme activity assay

Mannanase enzyme activity assay was performed using locust bean gum as substrate. The substrate was suspended in 50 mM Sodium citrate buffer pH 5.0 and heated until boiling while stirring continuously. The substrate was cooled and allowed to homogenize while stirring overnight. The insoluble material was removed by centrifugation [16] and supernatant was used as substrate for the reaction. Increasing concentration of total soluble protein (TSP, 10 µg to 100 µg) from cpMan and rMan were taken in a 500 µl reaction containing 0.5% locust bean gum substrate in 50 mM Sodium citrate buffer pH 5.0 at 70°C for 16 hours. BSA (100 µg/ml) was added to all reactions. Protein extract from untransformed plant and *E.coli* harboring pLD vector (without *man*1 gene) were used as negative controls. The reducing sugars released after the reaction were quantified by DNS method taking appropriate dilutions of the reaction samples. Absorbance was read at 540 nm and mannose was used as standard to measure the reducing sugars liberated after the reaction [61]. For mannanase unit calculation, carob galactomannan (Megazyme) was used as substrate. Mannanase activity in plants was quantified by comparison with enzyme activity of commercially available mannanase (Megazyme E-BMANN). One unit of mannanase activity is defined as the amount of enzyme which released one micromole of reducing-sugar equivalents per minute from low viscosity carob galactomannan (2 mg/ml, pH 5.0, and 70°C). All experiments had appropriate controls containing substrate without enzyme or enzyme without substrate.

### Hydrolysis of pinewood with chloroplast-derived enzymes

Dried pinewood sample (*Pinus ponderosa*) was obtained from KL Energy Corporation (Rapid City, SD, USA). Pinewood hydrolysis was carried out as described earlier [42]. Crude enzyme extracts obtained from the tobacco plants expressing a variety of biomass degrading enzymes were used to make various formulations of enzyme cocktails for pinewood hydrolysis. Prior to hydrolysis reaction, pinewood biomass was washed several times in distilled water until there was no sugar detected in the sample by DNS method. The hydrolysis reaction was carried out at 40–50°C for 36 hours in 50 mM sodium citrate buffer (pH 5.0), 5 mM CaCl_2_ and 100 µg of BSA per 5 ml reaction containing 200 mg pinewood. Pinewood hydrolysis was done at 50°C as other enzymes present in the cocktail were more active within the range of 40–60°C. Mannanase retained most of its activity at 50°C. The various cocktails were comprised of chloroplast-derived enzymes Mannanase (Man), Xylanase (Xyn gene from *Trichoderma reesei*), Cellulase (CelD gene from *Clostridium thermocellum*), Endoglucanase (Eg1 gene from *Trichoderma reesei*), Exoglucanase (CelO gene from *Clostridium thermocellum*), β glucosidase (Bgl gene from *Trichoderma reesei*), Pectate Lyases (Pel A, B, D genes from *Fusarium solani*), Acetyl xylan esterase (Axe1 gene from *Trichoderma reesei*) and Swollenin (Swo gene from *Trichoderma reesei*) [42]. The enzyme activity (units/mg) in crude total soluble protein of all chloroplast derived enzymes except mannanase was determined in earlier study [42]. In this study, for all cocktails, 200 µg TSP of each enzyme extract was used whereas negative control reaction contained 2000 µg TSP of untransformed leaf extract in 5 ml of hydrolysis reaction. All the reactions were carried out in a rotary shaker at 150 rpm. End product fermentable sugars were determined by DNS method [61] with D-glucose as standard. The percent hydrolysis of pinewood by different cocktails was calculated based on considering maximum release of fermentable sugars as 100% hydrolysis. The “percent increase” among other cocktails was calculated based on the release of sugars. Ampicillin and kanamycin 100 µg/ml was supplemented to inhibit any microbial growth during the prolonged hours of enzyme hydrolysis. All experiments were carried out in triplicate and statistical analysis was performed using t-test.
